# The Effects of Speed-Modulated Visual Stimuli Seen through Smart Glasses on Work Efficiency after Viewing †

**DOI:** 10.3390/s22062272

**Published:** 2022-03-15

**Authors:** Eiichi Hasegawa, Naoya Isoyama, Diego Vilela Monteiro, Nobuchika Sakata, Kiyoshi Kiyokawa

**Affiliations:** 1 Graduate School of Science and Technology, Nara Institute of Science and Technology, 8916-5 Takayamacho, Ikoma 630-0192, Nara, Japan; hasegawa.eiichi.gw1@is.naist.jp (E.H.); kiyo@is.naist.jp (K.K.); 2 Faculty of Computing, Engineering and the Built Environment, Birmingham City University, 1 Curzon Street, Birmingham B4 7XG, UK; diego.vilelamonteiro@bcu.ac.uk; 3 Faculty of Advanced Science and Technology, Ryukoku University, 1-5 Yokotani, Seta Oe-cho, Otsu 520-2194, Shiga, Japan; sakata@rins.ryukoku.ac.jp

**Keywords:** smart glasses, wearable computing, work efficiency, subjective time, after effects

## Abstract

It is known that subjective time and work efficiency are affected by visual stimuli. However, existing studies only consider the effects of visual information on the user during viewing and ignore the after effects. Using smart glasses lets users see visual information while moving until just before arriving at the office or school. We hypothesize that the user’s effects from the visual information they were looking at just before working or studying affects the subsequent work. Through two user studies, we investigated whether information presented on smart glasses affected subsequent work efficiency. In the first experiment, participants were presented with avatars running at two levels of speed, or no avatars, through simulated smart glasses in a virtual environment. They then solved a dot-clicking task on a desktop monitor. In the second experiment, we investigated whether the same effect could be shown while walking in the real environment, with a running and a fast-walking avatar both at the same speed in order to see the difference in the effects of the different movements. In the first experiment, we confirmed that the speed of later work tended to improve when presenting the running human-shaped avatar. From the results of the second experiment, which was conducted in the real environment, we did not confirm that the subsequent work speed varied depending on the type of avatar being displayed. As a reason for the trend of improvement in the task efficiency in the first experiment, observation of fast human motion may have unconsciously accelerated the observers’ body movement speed due to the mirror neuron mechanism. As a reason for why the work speed did not improve in the second experiment, the participants may be affected by other pedestrians and running cars. Additionally, it was difficult to see the images on the smart glasses while walking in the real environment.

## 1. Introduction

Smart glasses allow users to see visual information in various situations in a wearable computing environment. The smart glasses in this study are those that have a display in front of the users’ eyes and allow them to see the images. The users can see visual information displayed on the smart glasses while walking or riding a bicycle. In addition, they can see the information while commuting to work or school. In other words, by using smart glasses, users can see visual information while moving until just before arriving at the office or school.

The effects of what we see on our behavior and psychology have been studied for a long time. However, there has not been enough research on the effects of viewing specific information while performing some action or on the effects after viewing specific information. By clarifying the impact of visual information displayed on smart glasses on later behavior, we can expect effects such as improving the performance of later behavior simply by casually viewing information while commuting to work or school.

Some studies have been conducted on the effects of visual information displayed on smart glasses. They have confirmed that there are effects on human behavior. Shimizu et al., for example, proposed a system that presents visual stimuli that move around the periphery of the field of view on smart glasses and controls the user’s subjective speed of time according to the speed of the stimuli [1]. Isoyama et al. confirmed that a camera app icon displayed on smart glasses influences user’s behavior of photo-taking [2]. These studies are different from the objective of our study because they have investigated the effects related to the behaviors the users perform while looking at the visual information.

In this study, we investigate the effect of viewing information displayed on smart glasses while the user is performing some action on the user’s later behavior. We also consider how to make these positive effects for users. In this paper, we present a moving human-like avatar as the information presented on the smart glasses. We chose this because previous research has shown that people are affected by watching images of others displayed on a fixed monitor [3,4], and we believe that the same influence can be given to users by images displayed on smart glasses. In this study, we assume that the user can quickly undertake an action after viewing the image of a quickly moving avatar.

Primary contributions of the present paper are summarized as follows:

- To the best of our knowledge, this is the first paper that investigates the after effects of visual stimuli presented on smart glasses on the performance of subsequent tasks.
- Through two experiments, the effects of a running avatar presented to cycling participants with a head-mounted display on subsequent desk work were investigated, and it was found that the task performance was increased after viewing a running avatar.

The rest of the paper is organized as follows. We introduce related studies in Section 2. In Section 3, we explain our experiment on the effects of animation playback speed on moving users. Then, in Section 4, we verify whether the results obtained from the experiments in Section 3 hold up in the real environment. We discuss our approach and the experimental results in Section 5, and finally, in Section 6 we summarize our study.

Note that we have already introduced the first experiment in the poster paper [5]. The poster paper was a two-page manuscript and did not sufficiently describe our study. The experiment presented in the poster paper is the same as Experiment 1 in this paper. In this paper, we reconsider the results and discussion and add a second experiment.

## 2. Related Work

Various studies have shown that the speed of visual stimuli, such as video, affects the viewer’s perception and behavior (Table 1). It has been suggested that the effect is stronger when the visual stimulus for speed modulation contains a human. Watanabe created a video in which several moving black dots mimicked human motion and revealed that the reaction speed immediately after watching fast- and slow-motion movements became faster and slower, respectively [4]. The same trend was not observed after watching non-human motion, such as moving scattered points, suggesting that the timing of the observers’ action is synchronized with the speed of the human motion in the visual stimuli. It has also been shown that not only the actual speed of the movement in dynamic visual stimuli but also the speed of the movement implied in static visual stimuli affects subjective time. Nather et al. showed that subjective time was longer when viewing a still image of a person, which implied clear movement, than viewing a still image of a person, which implied little movement [6]. In contrast to Nather et al., Yamamoto et al. found that an image of abstract blocks imitating a human had a similar effect but did not affect subjective time if it was not perceived as a human [7]. These results are associated with mirror neurons, which provide a unified mechanism for observing and understanding a behavior and actually performing the behavior [8]. By observing the behavior of others with mirror neurons, it is thought that unconscious changes occur in the behavior of the observer. This effect has been shown to occur even when the observing and performing movements are different [4,9].

In the experiments of this paper, we will investigate whether the speed at which a person performs a task increases after being presented with a visual stimulus of fast movements. In this case, if the subjective time is changed, it is thought that the work speed will also be affected. If the subject feels that time is passing faster, we can expect that their actions will also be faster. Subjective time dilates and constricts when watching a fast video and a slow video, respectively [15]. Similarly, when watching a simple visual stimulus such as a walking stick figure on a monitor, the subjective speed of time is slower or faster as the speed of the stimulus becomes faster or slower, respectively [11,12,13,14]. The phenomenon that a filled interval with many events is perceived to be longer than an empty interval of the same physical duration is called the filled-duration illusion (FDI) [17,18,19]. Naturally, such illusions of subjective time become smaller or disappear with the smaller change in the speed of the visual stimuli [11,20]. Furthermore, it has been shown that the modulated subjective time induced by visual stimuli can affect the user’s work efficiency. Morton has demonstrated that the time to exhaustion becomes longer than normal when a slow-paced digital clock is presented while cycling on an ergometer [3]. Ban et al. have shown experimentally that displaying a fast-paced analog clock on a monitor increases working speed [16]. In these studies, users may have unconsciously changed the speed of body motion according to the perceived speed of time.

In this way, various studies have shown that user’s subjective time is manipulated by observing speed-modulated visual stimuli, specifically that of human motion. In most of the above-mentioned studies, the effect is investigated during the presentation of the visual stimuli. In a conventional VDT (Visual Display Terminals) working environment, the monitor is only viewed during work. Smart glasses, on the other hand, offer new opportunities where the user views visual information any time, such as while resting and exercising, and then starts working immediately. However, there exist few studies on how user behavior changes *after* viewing speed-modulated visual information. If we can show that work efficiency can be increased by viewing certain visual information in advance, then we can take advantage of such effects for improving the productivity of our daily work. In addition, proving the opposite effects will be helpful for deeper relaxation and behavioral restraint. In this study, we investigate whether viewing speed-modulated visual information while resting and exercising affects subsequent work efficiency.

## 3. Experiment 1: On the Effects of a Running Avatar on Exercising Users (Virtual Environment)

### 3.1. Method

#### 3.1.1. Hypotheses

In Experiment 1, we present cycling participants with a running avatar (3.00 steps/s), a fast-running avatar (5.25 steps/s), or no avatars, through simulated smart glasses in a virtual environment, and ask them to perform a dot-clicking task on a desktop monitor. The hypotheses to be tested in this experiment are as follows.

**Hypothesis** **1.** *Subjective time dilates with the presence of a running avatar, and the subsequent task performance increases.*

**Hypothesis** **2.** *Subjective time dilates further with a fast-running avatar, and the subsequent task performance increases further.*

#### 3.1.2. Setup

**Figure 1:**
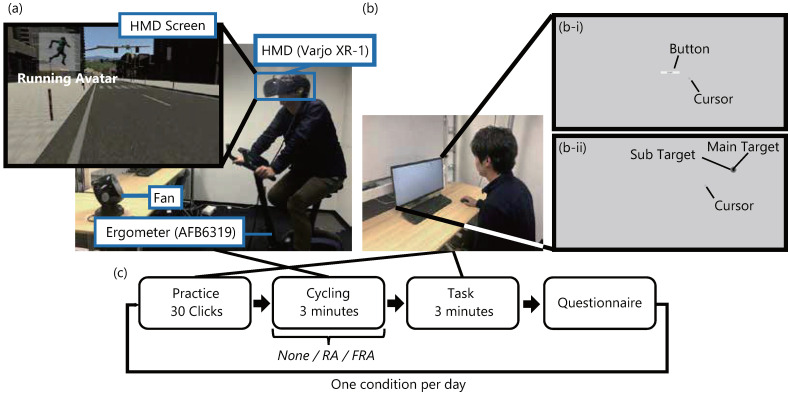
(**a**) A virtual cycling system; (**b**) Tasks after cycling; (**b-i**) Elapsed time-estimation task; (**b-ii**) Dot-clicking task; (**c**) Procedure of Experiment 1.

We focus on the effect of the images displayed on the smart glasses that the user sees while moving. In Experiment 1, we assume that the user is pedaling a bicycle. Referring to the previous studies [21,22], we used an immersive virtual environment to simulate an outdoor cycling situation with consistent traffic and weather (see Figure 1a). A head-mounted display (Varjo XR-1) and an ergometer (ALINCO AFB6319) were used for the virtual cycling experience.

Participants pedal and drive the ergometer on a flat, straight street in a virtual city designed using Unity. As haptic feedback to improve the reality of cycling, a fan was also used to blow the wind from the participants’ frontal direction.

The velocity of the virtual bicycle is set to 19.5 [km/h] and maintained regardless of the physical pedaling tempo, because the speed at which the landscape changes may affect the subsequent task performance. This velocity was determined as the average speed of 10 participants in the preliminary study who were asked to pedal for three minutes while imagining that they were cycling in the city at their normal tempo on their way to school or for shopping.

**Figure 2:**
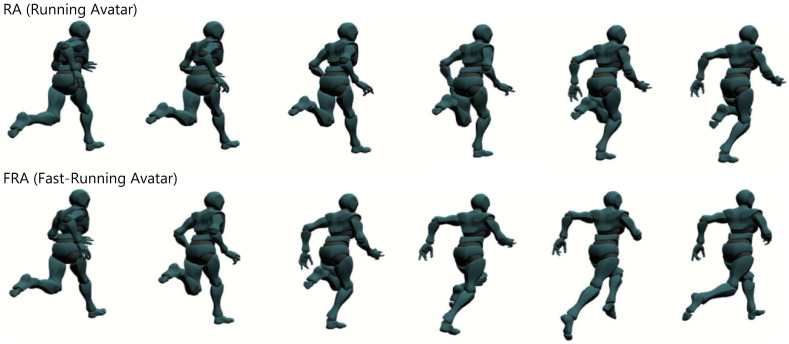
The appearance of each avatar (per frame).

In this experiment, we compare three conditions with regard to a running avatar. In two conditions out of three, a running avatar is always presented at the top left corner of the field of view of the simulated smart glasses. The displayed avatar stays at the same place and in the same orientation in the participant’s head coordinate system. When the participants move their heads, the displayed avatar follows the movement. In these conditions, the avatar is running at two different levels of speed facing the frontal direction rotated 30${}^{\circ }$ to the right; a speed of jogging (3.00 [steps/s], *RA (Running Avatar)* condition) and a speed of sprint (5.25 [steps/s], *FRA (Fast-Running Avatar)* condition). The appearance of each avatar is shown in Figure 2. The running speed in the FRA condition was determined by three participants in the preliminary experiment as the fastest speed that could be recognized as running when presented in the periphery of the field of view. The avatar orientation was also determined by the same preliminary experiment as the easiest orientation in which to recognize the movement. On the other hand, no avatar is presented in the *None* condition for comparison. After the virtual cycling, participants sit on the chair in front of the desk with the desktop monitor and perform the elapsed time estimation task and the dot clicking task, which we will explain below.

#### 3.1.3. Procedure

Figure 1c shows the procedure of Experiment 1. First, in order to reduce the learning effects, participants are seated in the chair and perform both the elapsed time-estimation task and the dot-clicking task for 30 targets using the desktop monitor before the virtual cycling. These practice tasks are the same as the main tasks (Figure 1b) after the virtual cycling.

The elapsed time-estimation task (Figure 1(b-i)) measures the subjective speed of time. Participants first click on a button on the monitor to start the elapsed time estimation task. They click on the button again when they feel that 10 seconds have passed. Quantitative measurement of the subjective speed of time after the virtual cycling helps us to investigate the mechanism of change in the task performance, if it is found.

The completion of the elapsed time-estimation task initiates the dot-clicking task. The dot-clicking task requires the participants to click on circular targets that appear one after another at random locations on the desktop monitor (Figure 1(b-ii)). The target is two-layered with an outer gray disc with a radius of 7.0 mm and an inner black disc with a radius of 2.5 mm. Participants are instructed to click on the target as quickly and accurately as possible. Clicking inside the outer target is enough to proceed to the next target, but they are encouraged to click on the inner target as much as possible. A new target appears at a random location by clicking on the current target, but it is at least 70 mm away from the current one. We assume an environment where a user moves to his/her office while seeing images on the smart glasses and works at the destination after arriving. We hypothesize that the work speed will increase after seeing the avatar running fast, and we expect the number of clicks to increase in this experiment.

After these two practice tasks, participants ride on an ergometer wearing the HMD and earplugs. At the beginning of the virtual cycling (Figure 1a), an instruction label “Faster” or “Slower” is shown on the HMD screen if they pedal too slow or fast, respectively, in order to help them pedal at a specified pace. When they pedal at the specified pace for 10 consecutive revolutions, no further instruction is shown and the virtual scenery starts to flow in one of the three avatar conditions. After that, they are asked to pedal in the virtual city for three minutes, keeping in mind to maintain the initial speed. They are instructed not to look around more than necessary to simulate user’s view of real cycling in the city. They are not informed on how the avatar conditions differ from one another, nor the names of each condition.

After cycling, participants take off the HMD and the earplugs and perform the main elapsed time-estimation task for about 10 seconds and the main dot clicking task for three minutes on the desktop monitor. After the tasks, they answer a questionnaire consisting of four items for the virtual cycling and five items for the main dot clicking task each on a 7-point Likert scale (see Table 2).

The above-mentioned procedure is repeated three times for the different avatar conditions. Each participant conducts only one condition per day to prevent potential boredom and fatigue affecting the results. The order of the avatar conditions is counterbalanced between participants based on a Latin square design.

The number of pedal revolutions, the actual elapsed time to estimate 10 seconds, the number of clicks on the (outer) target, the success rates (the percentage of clicks on the inner target), and the subjective ratings are recorded and analyzed.

#### 3.1.4. Participants

In total, 18 participants (9 males and 9 females) aged between 22 and 27 took part in Experiment 1. They were asked to refrain from eating and exercising for 30 min before the beginning of the experiment. They were told that the experiment was designed to see how they would be affected by the visual stimuli. They were not told what effects were expected to occur in detail.

### 3.2. Results

**Figure 3:**
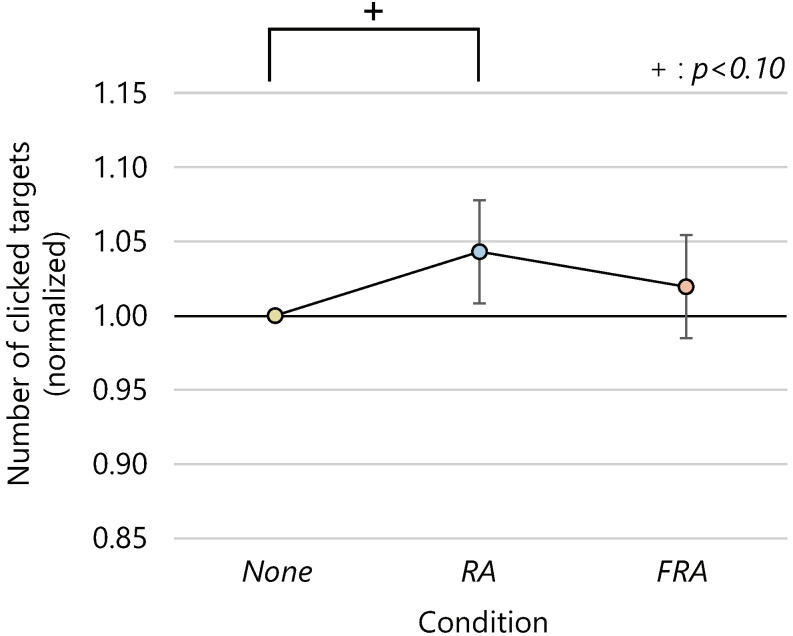
Average number of clicked targets (normalized).

The average numbers of clicked targets and the 95% confidence intervals were 131.1 ± 5.6, 136.3 ± 5.2, and 133.4 ± 6.7 in the *None*, *RA*, and *FRA* conditions, respectively. The number of clicked targets was the highest in the *RA* condition for 10 out of 18 participants. Figure 3 shows the average number of clicked targets normalized to 1.0 for the number of clicked targets in the *None* condition. An ANOVA revealed a significant difference in the normalized average of the number of clicked targets for the effect of presence or absence of running humanoid avatars and speed on clicks (p<0.05). A post-hoc analysis by Wilcoxon signed-rank test with the BH method found significant tendency between the *None* and the *RA* conditions (p<0.10). Although there was no significant difference between the *FRA* condition and the other conditions, the mean number of clicks in the *FRA* condition was located between the other conditions.

**Figure 4:**
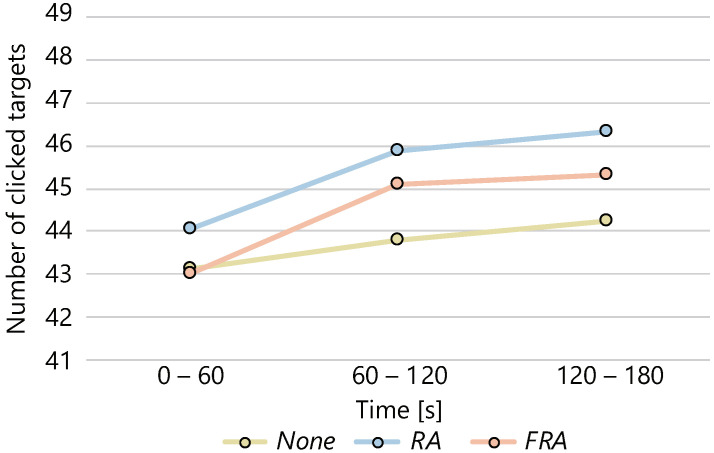
Changes in the number of clicks over time.

**Figure 5:**
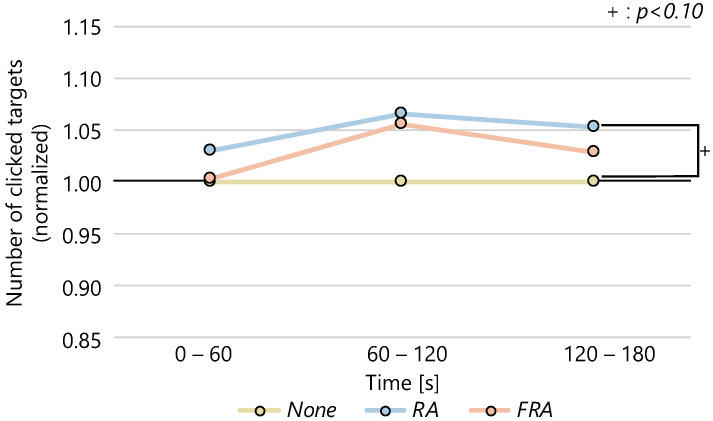
Changes in the number of clicks over time (normalized).

The average number of clicks in each condition every 60 s is shown in Figure 4, and the average of the normalized values is shown in Figure 5. The ANOVA was performed on the difference in the number of clicks for each condition at each time and the change in the number of clicks over time between conditions. A significant difference was found between the three conditions (p<0.05) in the number of clicks for each condition in the interval 60–120 s, normalized with respect to the *None* condition. Although we conducted multiple comparisons, no significant differences were found among the conditions. In the interval 120–180 s, the ANOVA showed a significant tendency among the three conditions. We conducted multiple comparisons and confirmed the significant tendency among the *None* and the *RA* conditions (p<0.10).

**Figure 6:**
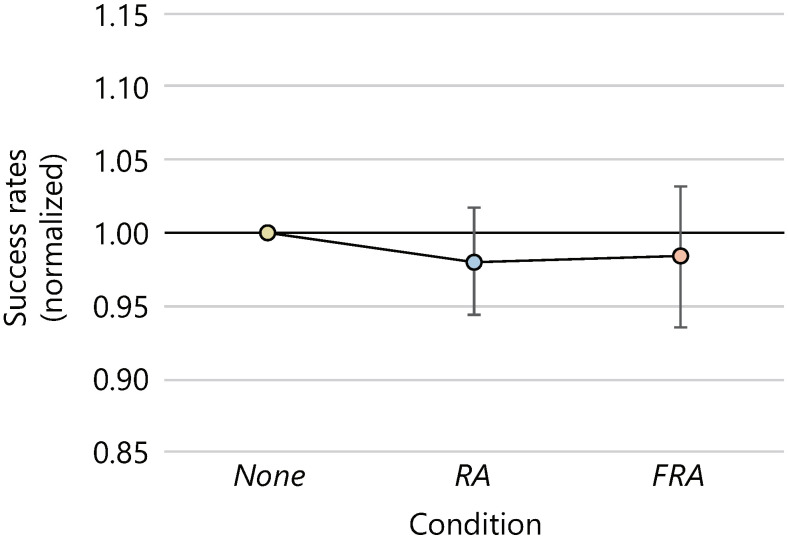
Average success rates (normalized).

The mean success rates (the percentage of the clicked inner targets) and the 95% confidence intervals were 90.3 ± 2.8 [%], 88.4 ± 3.4 [%], and 88.8 ± 4.7 [%] in the *None*, *RA*, and *FRA* conditions, respectively. Figure 6 shows the average success rates normalized by the z-score normalization. An ANOVA found no significant differences in the normalized average success rates between the three avatar conditions.

The mean actual elapsed time to estimate 10 seconds were 11.2 ± 0.8 s, 11.1 ± 0.7 s, and 11.3 ± 0.5 s in the *None*, *RA*, and *FRA* conditions, respectively. An ANOVA found no significant differences in the mean elapsed time between the three avatar conditions.

**Figure 7:**
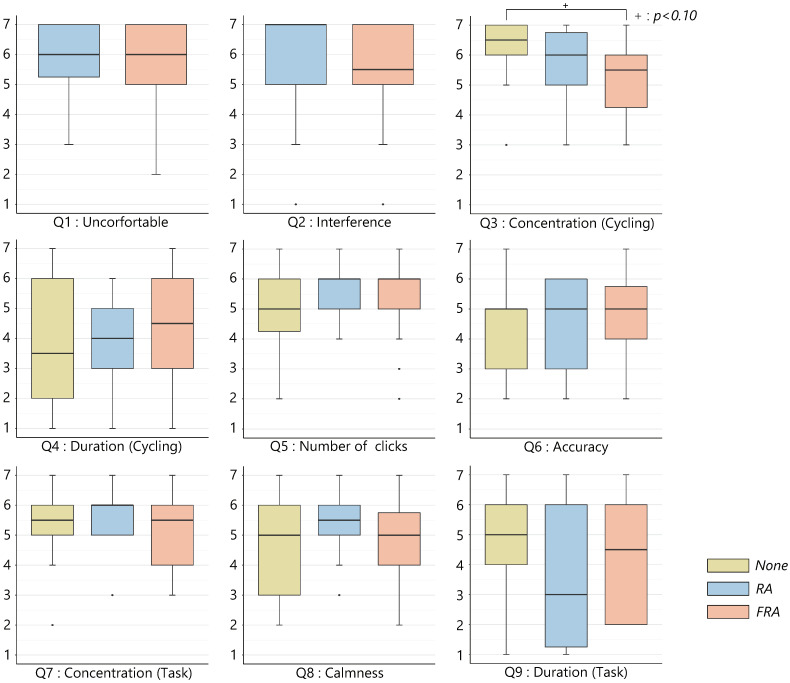
Subjective assessment (Experiment 1).

Figure 7 shows the results of the subjective assessment. The ratings of the subjective assessment are treated as an interval scale for convenience. An ANOVA using the ART revealed a significant difference in the questionnaire item on concentration (Q3) (p<0.05). Multiple comparisons with a Wilcoxon signed-rank sum test using the BH method (Benjamini–Hochberg) revealed a trend toward significance between the *None* and the *FRA* conditions (p<0.10). No significant differences were found in any of the other questionnaire results.

**Figure 8:**
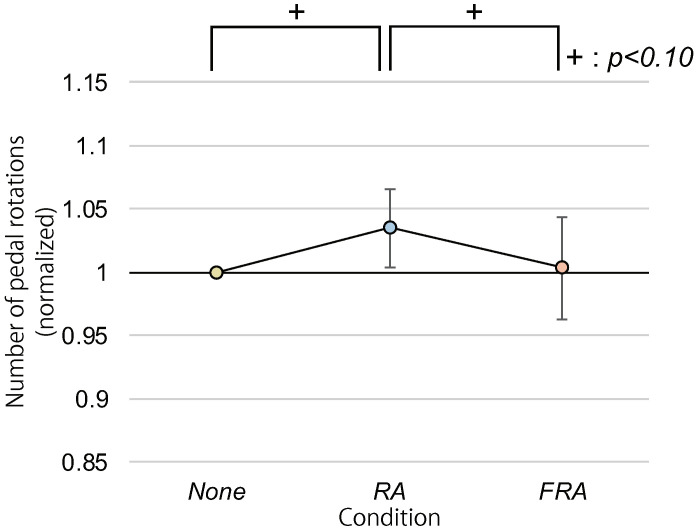
Average number of pedal revolutions (normalized).

The mean numbers of pedal revolutions and the 95% confidence intervals were 161.7 ± 6.5 [times], 167.1 ± 6.4 [times], and 161.7 ± 6.5 [times] in the *None*, *RA*, and *FRA* conditions, respectively. Figure 8 shows the mean numbers of pedal revolutions normalized by the z-score normalization. An ANOVA revealed a significant difference in the normalized numbers of revolutions between the three avatar condition (p<0.05). Multiple comparisons showed trends toward significance between the *RA* and the *FRA* conditions and the *None* and the *RA* conditions (p<0.10).

### 3.3. Discussion

In this experiment, we investigated the effects of the presence and speed of a running avatar appearing in the peripheral field of view of cycling participants on the subsequent work efficiency. In the dot-clicking task, a significant trend was found in the number of clicked targets between the *None* and the *RA* conditions. There was a tendency that the participants clicked on the targets more often in the *RA* condition compared with the *None* condition. Thus, Hypothesis 1 was partially supported. No significant differences were found in the elapsed time-estimation task, so the presence and speed of a running avatar were not proven to affect the subjective speed of time. As a reason for the trend of improvement in the task efficiency in the case of the *RA* condition, observation of fast human motion may have unconsciously accelerated the observers’ body movement speed due to the mirror neuron mechanism [8].

As evidence of the influenced speed of body movement, there was also a tendency that the pedaling speed was faster in the *RA* condition compared with the other conditions. The participants’ movement tended to be quicker when observing a running avatar, and this in itself may have contributed to improving the performance of the subsequent dot-clicking task. In the future, we will use a treadmill to fully control the speed of movement of participants and investigate the effects of visual stimuli solely on mental performance without the influence of physical performance.

Hypothesis 2 was rejected because no further performance gain was observed when the sprint avatar was presented in the *FRA* condition. The pedaling speed in the *FRA* condition was actually slower in average compared with the *RA* condition. Unlike the *RA* condition, the participants’ body movements were not entrained to the speed of the sprint avatar in the *FRA* condition. One possible reason for this is that the speed of sprint avatar was too fast so that the participants may have given up adjusting the speed of their movements.

No significant differences were found in the success rates and the questionnaire item on calmness (Q8) between the three avatar conditions. The success rates did not decline even when the number of clicked targets increased in the *RA* condition. These results suggest that presenting an avatar running at the speed of jogging accelerates the user’s body motion and increases the performance of subsequent tasks without sacrificing a sense of calm. Visual stimuli in the RA condition seem appropriate to present on smart glasses while cycling before work. On the other hand, the results of the questionnaire item on concentration (Q3) suggest that the participants could not concentrate on cycling well in the *FRA* condition compared with the *None* condition. Visual stimuli in the *FRA* condition seem distracting and inappropriate to present on smart glasses while cycling before work.

## 4. Experiment 2: On the Effects of a Running Avatar on Exercising Users (Real Environment)

The experiment results in Experiment 1, conducted in the virtual environment, suggested that users who watched the animation of the running human avatar during exercise improved their work speed later. We can expect similar effects in the real environment. However, the virtual environment used in Experiment 1 ignores distracting objects and information such as turning corners and other pedestrians, and any environmental noise. In order to claim the effect of avatars and to apply it as a system, it is necessary to confirm the effect in the real environment. Therefore, in this section, we verify whether the presentation of a human-shaped avatar in the peripheral vision affects the speed at which participants perform later tasks in the real environment. In Experiment 1, we examined the effects during cycling. However, some manufacturers of smart glasses prohibit wearing them while cycling, so in this section, we examine the effects during walking in the real environment.

### 4.1. Method

#### 4.1.1. Hypotheses

The results of Experiment 1 suggest that when participants see the running human avatar presented during exercise in the virtual environment, the participants’ subsequent work speed tended to be improved. Similarly, in the real environment, by observing the quick movements of others, it is expected that we unconsciously synchronize our later actions and improve our work speed. The effects of mirror neurons on subjective time and reaction time have been shown to be stronger when the movement being observed is similar to the movement being performed. There is a possibility that the effect of mirror neurons increases by presenting avatars that walk fast rather than avatars that run when walking. The hypotheses to be tested in this experiment are as follows.

**Hypothesis** **3.** *The presentation of a fast-acting avatar unconsciously increases the work speed of the participant after walking.*

**Hypothesis** **4.** *If the avatar movements are similar to the movements the participant is performing, the work speed is increased.*

By showing the differences in the effects of different types of avatar movements, it will be possible to present the avatar appropriately according to the situation. In the following, we describe the experiments we conducted to test our hypotheses.

#### 4.1.2. Setup

**Figure 9:**
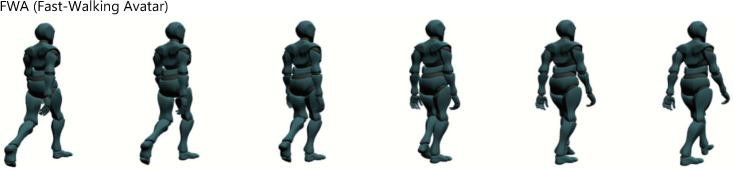
Appearance of a fast-walking avatar (per frame).

In order to test the hypotheses, the same human-shaped avatar as in Experiment 1 is presented in the participant’s field of view while he/she is walking. Two types of avatars were used; an avatar running at a speed similar to the running speed used in Experiment 1 (3.00 [steps/s], *RA* (Running Avatar) condition), and an avatar walking fast at the same speed (*FWA* (Fast-Walking Avatar), Figure 9). The avatar is presented on the smart glasses (MOVERIO BT-300) and it keeps running in a fixed position in the upper left corner of the participant’s field of view, facing a direction rotated 30 degrees to the right from the front. In order to clarify the effect of the avatar presentation, we also prepared a condition in which nothing is presented on the smart glasses (*None* condition), as in Experiment 1, and compared the three conditions.

**Figure 10:**
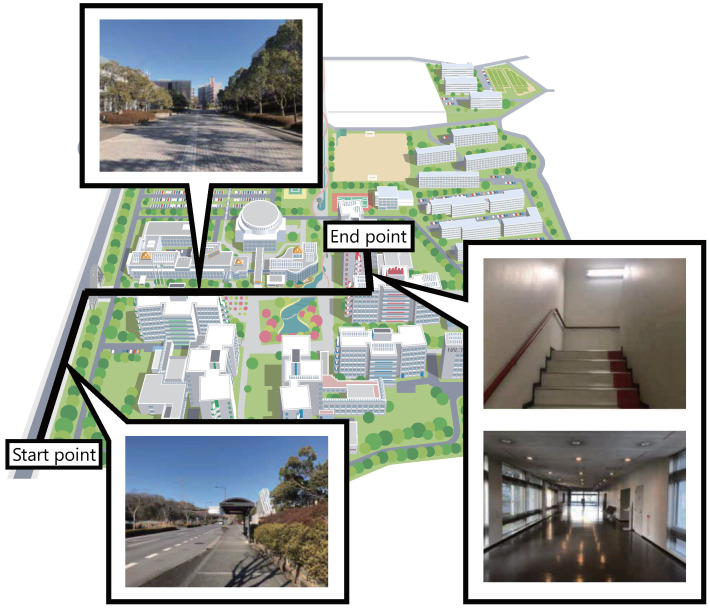
The course in Experiment 2.

The participants walk a course (Figure 10) of about 500 m from the outside of the university to the laboratory (a designated room in the university). The time required for the walk is approximately seven minutes. The participants memorize the course before the experiment. In the experiment, the experimenter follows the participant from behind. The experimenter instructs the participant to turn at designated locations. After the walk, in order to evaluate the differences in the effects of the different conditions, the participants perform the same time-estimation task (Figure 1(b-i)) and the same clicking task (Figure 1(b-ii)) as Experiment 1.

#### 4.1.3. Procedure

Each participant takes part in the three conditions separately on independent days. On each day, the participants perform a practice task and then walk a course specified by the experimenter. After the walk, they perform the tasks and answer questionnaires.

Before each walk, the participants perform a time-estimation task and a 3-minute clicking task as a practice task, which will be used in the experiment. These tasks are the same as in Experiment 1. In Experiment 1, the number of clicks in the first trial was significantly lower than in the second and third trials, regardless of the condition. Therefore, we set a longer practice time for the participants to get used to the clicking task. They can click approximately 120 times in three minutes.

**Figure 11:**
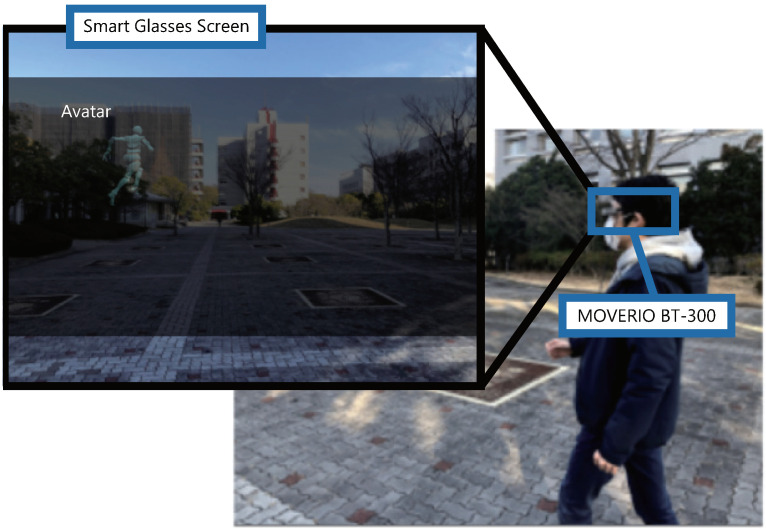
Walking while wearing smart glasses.

At the beginning of the walk, the participants wear smart glasses and walk with one of the conditions presented to them from the starting point outside the university campus (Figure 11). The order of the conditions was changed for each participant according to a Latin square design.

After arriving at the experimental room (End point in Figure 10), the participants take off the smart glasses and perform a time-estimation task and a 3-minute clicking task on a fixed monitor. After completing the tasks, the participants answer a 7-point subjective evaluation questionnaire consisting of four subjective evaluations of walking and five subjective evaluations of the clicking task, as shown in Table 2.

We do not explain the difference in speed of the running avatars to the participants, and we do not tell them the name of the condition. After the experiment, we compared the number of clicks on the target in the task, the success rate (the percentage of clicks on the inner black circle), the time evaluation (when the participant felt that 10 s had passed), and the subjective evaluation.

#### 4.1.4. Participants

The experiment was conducted on 12 participants (10 males and 2 females) between the ages of 22 and 27. As in Experiment 1, the participants were asked to refrain from eating or exercising immediately before the start of the experiment. They were told that the experiment was designed to see how they would be affected by the visual stimuli. They were not told what effects were expected to occur in detail.

### 4.2. Results

**Figure 12:**
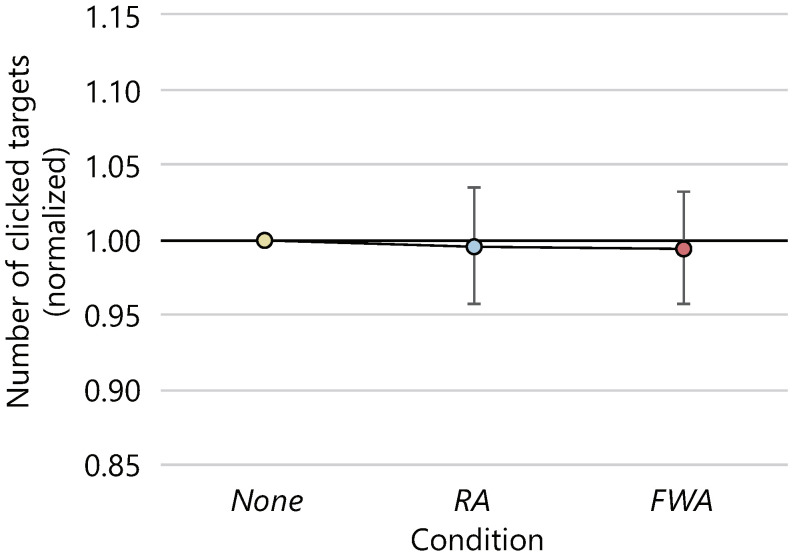
Average number of clicked targets (normalized).

**Figure 13:**
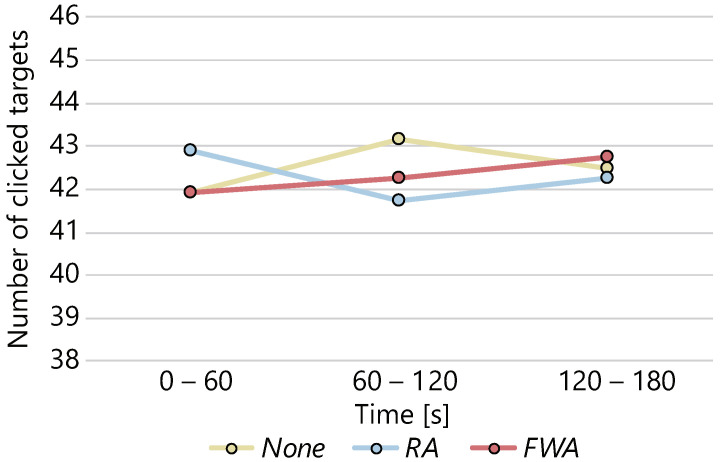
Changes in the number of clicks over time.

The average numbers of clicked targets and the 95% confidence intervals were 127.6 ± 6.5, 126.9 ± 7.6, and 126.7.4 ± 7.4 in the *None*, *RA*, and *FWA* conditions, respectively. Figure 12 shows the average number of clicked targets normalized to 1.0 for the number of clicked targets in the *None* condition. The ANOVA was conducted on the number of clicks, but the effect of the presence or absence of avatars and the difference in motion on the number of clicks could not be confirmed with statistical significance. The average number of clicks for each condition every 60 s is shown in Figure 13. There was no statistical difference in the number of clicks between conditions at each interval, nor in the change in the number of clicks over time between conditions.

**Figure 14:**
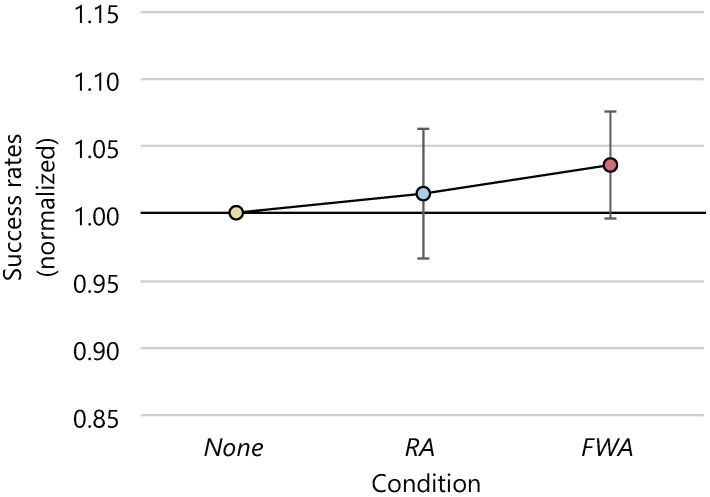
Average success rates (normalized).

The average success rate of the task and the 95% confidence intervals were 86.1 ± 4.3 [%], 87.6 ± 6.3 [%] and 88.8 ± 3.6 [%] in the *None*, *RA*, and *FWA* conditions, respectively. Figure 14 shows the result of normalizing the success rate for each participant. The ANOVA was performed on the success rates, but no statistically significant differences were found between the conditions.

The average value of time evaluation in the time estimation task before the start of the clicking task and the 95% confidence intervals were 11.2 ± 0.5 s, 11.3 ± 0.6 s and 11.4 ± 0.7 s in the *None*, *RA*, and *FWA* conditions, respectively. There were no statistically significant differences between the conditions.

**Figure 15:**
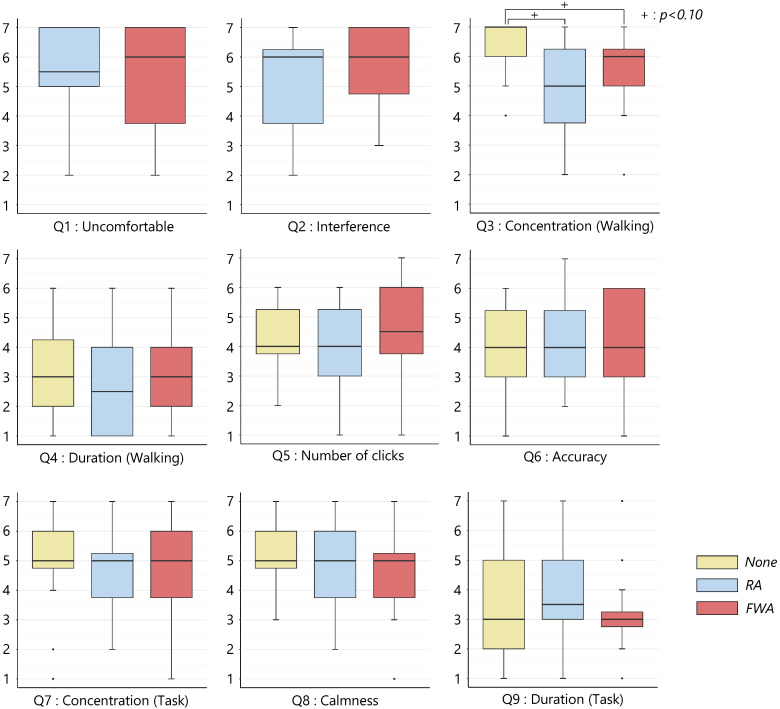
Subjective assessment (Experiment 2).

Figure 15 shows the results of the subjective evaluation answered after the experiment in each condition. The ANOVA using the ART confirmed a significant difference (p<0.05) for the item Q3 “I could concentrate on walking.”. As a result of post-hoc analysis by Wilcoxon signed-rank test with the BH method, we were able to confirm a significant tendency between the *None* and *RA* conditions and between the *None* and *FWA* conditions (p<0.10). There were no significant differences in the other items between the conditions.

### 4.3. Discussion

Based on Experiment 1, we experimented with investigating whether the speed of later work is changed by presenting a fast-moving human-shaped avatar on the smart glasses when walking in the real environment. At the same time, we examined whether the effects differed depending on the type of movement of the avatars, such as running or fast walking. From the results of the clicking task performed after walking, we could not confirm any difference in the number of clicks with or without avatars or with different movements. Therefore, Hypotheses 3 and 4 were rejected. Experiment 1, in which participants rowed an ergometer in the virtual environment, suggested that the presentation of the running avatars tended to improve the speed of later work. On the other hand, such a tendency could not be confirmed in this experiment. One of the reasons for this result was that the avatars were not as clear in the real environment as in the virtual environment. In the experiment in the virtual environment, the avatars were not semi-transparent and were clearly visible in the participant’s field of vision. However, because the smart glasses used in this experiment were see-through, the presented avatars were semi-transparent and were difficult to see, especially outdoors. In addition, the participants did not need to be aware of the surrounding environment because the bicycle riding in the virtual environment was just pedaling along a straight route. In this experiment, the participants had to be aware of things other than avatars in their field of vision, such as turning corners, other pedestrians, and cars.

For the subjective evaluation item Q3, “I could concentrate on walking.” the values in the *RA* and *FWA* conditions tended to be lower than those in the *None* condition, suggesting that the participants were not able to concentrate on walking due to the presentation of the avatars. Therefore, the presentation of avatars during walking is considered inappropriate because it distracts the user.

## 5. Discussion

In this study, we investigated whether the visual information presented on smart glasses in the exercising situation affects the performance of the subsequent tasks. Experimental results showed that the speed of the subsequent tasks changed even after the presentation of visual information has been finished.

In Experiment 1 in Section 3, we confirmed that viewing the running avatar at the speed of jogging while cycling tends to accelerate the pedaling speed and the subsequent task. No difference was found in the subjective speed of time compared with the condition with no avatar, suggesting that the improved performance was due to the mirror neuron mechanism. Assuming that these effects are maintained in real cycling situations, it can be expected that work efficiency will be increased by presenting similar visual stimuli during pre-work cycling.

In Experiment 2 in Section 4, based on Experiment 1, in which participants were made to pedal an ergometer in the virtual environment, we examined whether the same tendency could be shown by walking in the real environment. However, we could not confirm the effect of presenting a running or fast-walking human-shaped avatar on the participants’ work speed. In the experimental environment of Experiment 2, other pedestrians were walking, and cars were running. While the participants walked along the course, they watched the animation on the smart glasses and saw other pedestrians. We thought it would be worthwhile to experiment in an environment that simulates an actual use case, instead of the experiment in which the landscape only changes and there are no other pedestrians, as in Experiment 1. In the actual use case, the participants may be affected by other pedestrians. On the other hand, since the participants can always see the image displayed on the smart glasses, we thought that the effect of the image might be stronger than the effect of the surrounding situation. However, in reality, the work speed did not change after watching, and we obtained different results from Experiment 1. In this experiment, we did not record the participants’ surroundings. In addition, we did not record how often the participants looked at the avatar. In the future, we will record these data and verify what factors reduced the effect and how to present the avatar to obtain the effect as in Experiment 1. The results of Experiment 2 show that simply presenting avatars does not produce the same results as in Experiment 1, which is useful information for future development.

The number of participants was 18 in Experiment 1 and 12 in Experiment 2, which are both small. For the results of the clicking task in Experiment 1, we calculated the power to be 0.33. To obtain a power of 0.80, we should have recruited 53 participants. For the results of the clicking task in Experiment 2, we calculated the power to be 0.05, which is a very low value. It should be noted that the results described in this paper are based on low power conditions.

We tested the presentation of visual stimuli in only two situations: cycling in a virtual city and walking in a university. However, actual user movements and the surrounding environments will be much more diverse. In the future, we will investigate the effects of various body movements and surrounding environments. In addition, we apply these findings to develop a wearable system that adaptively presents appropriate visual information depending on the context and schedule of the user, and controls user behavior accordingly.

Several issues need to be considered in order to develop a practical system. We used small visual stimuli in Experiment 1 to minimize the interference with the visibility of the scenery. However, some manufacturers of smart glasses prohibit the wearer from wearing them while riding a bicycle. We need to design an information-visualization policy for safe cycling. In addition, it is possible for some users that opposite effects may appear on the subsequent task performance compared to the general trends. Such a user will work more slowly when he or she actually wanted to work more quickly. In the future, we would like to investigate such individual differences and develop a personalized information-visualization method.

## 6. Conclusions

In this paper, we investigated the effect of viewing information displayed on smart glasses while the user is performing some action on the user’s later behavior. We presented a moving human-like avatar as the information presented in the smart glasses, and conducted two experiments. In the experiments, we compared the speed of work after the avatar was presented as visual information during motion.

In Experiment 1 in Section 3, we investigated the effect of the animation speed of the running human-shaped avatar on the user when exercising. In the experiment, we confirmed that the speed of later work tended to improve when the running human-shaped avatar was presented on the peripheral vision of a user in a motion state while riding a bicycle in the virtual environment. On the other hand, based on the mechanism of mirror neurons, we expected that the faster the speed of the avatars, the faster the work speed would be, but such an effect could not be confirmed.

In Experiment 2 in Section 4, based on the experiment in Section 3 using the ergometer in the virtual environment, we verified whether the same tendency could be shown in walking in the real environment. In addition, to verify the differences in the effects of the different types of avatars’ movements, we presented a running avatar and a fast-walking avatar to verify the differences in the effects. As a result, the effect of avatars on the work speed could not be confirmed. The difference in the visibility of the avatars in the virtual and real environments is thought to have made it difficult for the participants to be affected. Since it has been shown in Experiment 1 that the presentation of avatars tends to be effective in improving work speed, it is conceivable that the same tendency can be shown for users in a state of motion in the real environment, depending on the conditions. Therefore, one of the future tasks is to investigate the factors that prevented the effect and to improve the presentation method.

The results of the experiments in this paper can be applied to a system for controlling work speed. However, there are many issues that need to be investigated and overcome before the system can be realized, such as the duration of the improvement in work speed due to visual information, the effects of body movement and scenery when wearing smart glasses, and individual differences in the effects. Therefore, it is necessary to analyze the effect of visual information on work speed in more detail.

## Figures and Tables

**Table 1:** Comparison of related work that investigated the impacts of the speed of visual stimuli on different parameters.

	Effect				When		Display	
	Subjective Time	Work Efficiency	Reaction Speed	Exhaustion	During	After	Desktop Monitor	Smart Glasses
David M Eagleman et al. [10]	✓				✓		✓	
Hakan Karşılar et al. [11]	✓				✓		✓	
Sae Kaneko et al. [12]	✓				✓		✓	
Scott W Brown [13]	✓				✓		✓	
Joshua S Beckmann et al. [14]	✓				✓		✓	
Jeremy Grivel et al. [15]	✓					✓	✓	
Tomoyuki Shimizu et al. [1]	✓				✓			✓
Yuki Ban et al. [16]		✓			✓		✓	
Katsumi Watanabe [4]			✓		✓		✓	
R Hugh Morton [3]				✓	✓		✓	
Present work		✓				✓		✓

**Table 2:** Questionnaire items (Experiment 1, 2).

	Phase	Questionnaire Item
Q1		Avatar was NOT uncomfortable. (Uncomfortable)
Q2	[Exp. 1] Cycling	Avatar was NOT interfering. (Interference)
Q3	[Exp. 2] Walking	I could concentrate on cycling/walking. (Concentration)
Q4		I felt that the virtual cycling/walking was long. (Duration)
Q5	Task	I could click on a lot of targets. (Number of clicks)
Q6		I could click on the targets accurately. (Accuracy)
Q7		I could concentrate on clicking. (Concentration)
Q8		I could calmly click on the targets. (Calmness)
Q9		I felt that the dot clicking task was long. (Duration)

## Data Availability

The data presented in this study are available on request from the corresponding author.
